# Editorial: Reciprocal crosstalk between the tumor microenvironment and cancer stem cells

**DOI:** 10.3389/fimmu.2024.1505225

**Published:** 2024-11-05

**Authors:** Rosanna Paciucci, Amancio Carnero, Matilde Esther LLeonart

**Affiliations:** ^1^ Cell Signaling and Cancer Progression Laboratory, Vall d’Hebron Institute of Research (VHIR), Barcelona, Spain; ^2^ Department of Biochemistry and Molecular Biology, Autonomous University of Barcelona, Barcelona, Spain; ^3^ Instituto de Biomedicina de Sevilla (IBIS), Virgen del Rocio Hospital (HUVR), Consejo Superior de Investigaciones Científicas (CSIC)/Universidad de Sevilla, Seville, Spain; ^4^ Biomedical Research in Cancer Stem Cells Laboratory, Vall d’Hebron Institute of Research (VHIR), Barcelona, Spain

**Keywords:** cancer, cancer stem cells, microenvironment, resistance, immune system

The tumor microenvironment (TME) plays a pivotal role in cancer growth and treatment resistance, comprising not just the tumor cells themselves, but also a variety of cells, including T lymphocytes and cancer-associated fibroblasts (CAFs) among others. Within this dynamic setting, cancer stem cells (CSCs), a distinct subpopulation within tumors capable of self-renewal and differentiation, emerge as a resilient subpopulation that contributes significantly to tumor aggressiveness and relapse. CSCs exhibit several mechanisms that foster their survival, such as metabolic alterations and the ability to expel drugs, thereby complicating treatment efforts. The intricate interplay between the TME and CSCs is essential for cancer initiation and progression, influencing key processes like survival, niche formation, immune interactions, angiogenesis, and therapeutic resistance. In this sense, the following articles provide novel insights into how TMA-CSC interactions may be relevant in therapeutic aspects.

Brockmueller et al. investigate the effects of resveratrol, a plant-derived antioxidant, in colon cancer cells. Their study reveals that resveratrol can suppress cell viability in p53-proficient colon cancer cells while showing limited effectiveness in colon cancer cells lacking functional p53. Utilizing a three-dimensional model, they found that high concentrations of resveratrol (10-60 μM) notably reduced cell viability, migration, and plasticity in HCT-116 cells with functional p53, but not in p53-deficient variants. The mechanism appears to involve the inhibition of the deacetylase Sirt-1, that results in increased acetylation of p53. The hyperacetylated p53 promotes the release of cytochrome C and the activation of caspases, ultimately triggering a p53-driven apoptosis.

These findings suggest that resveratrol by modulating critical signaling pathways, could be a promising therapeutic agent targeting cancer cells within the colorectal cancer TME.

Zhang et al. focuses on the human APOBEC3 (A3) gene family, that encode enzymes that are part of the body’s innate immune system, that are known primarily for its role in immune defense against viral infections. However, these enzymes can also damage cellular DNA, potentially contributing to the onset and growth of cancer.

Their study highlights a correlation between the expression of A3s with increased mutational burden and microsatellite instability in bladder, breast, and colon cancer.

Specifically, A3C was positively associated with glioma stem cell markers, suggesting that A3C plays a significant role in maintaining CSCs and promoting tumor progression. Additionally, the authors established a predictive model indicating that A3 expression levels could serve as independent prognostic factors in glioma patients. Interestingly, GO and KEGG analyses suggested that A3C is involved in diseases related to the immune system. Higher levels of A3C appear to play a role in the formation of an immunosuppressive TME in glioma.

This study provides an initial framework for exploring the role of A3Cs in CSCs and the immune system and underscores A3 enzymes’ potential in shaping an immunosuppressive TME and their viability as therapeutic targets in cancer treatment.

Guha et al. describe the interactions between cancer stem cells (CSCs) and proteins of the TME related to immune cell roles in breast cancer. Tumor-associated macrophages (TAMs) promote tumor growth through cytokines while suppressing immune responses. Tumor-infiltrating neutrophils (TINs) can adopt either antitumor (N1) or protumor (N2) roles, influenced by signals like TGF-β. Eosinophils can attract CD8+ T cells and induce inflammation but may also facilitate tumor progression through factors like MMP-9 and VEGF. In the adaptive immune response, T cells, particularly CD8+ cytotoxic T cells, are crucial, though their effectiveness declines with cancer progression. Breast CSCs produce TGF-β, suppressing T-cell activity and promoting immune tolerance through PD-L1 expression, especially in triple-negative breast cancers. Tumor-infiltrating lymphocytes (TILs) are heterogeneous, with low TIL levels leading to poor immunotherapy responses. CSCs evade immune surveillance via immunosuppressive factors and MHC downregulation.

Recent therapies, including targeting BCSC markers like CD44 and CD133 and combining antibody therapies with chemotherapy, show promise in overcoming treatment resistance and improving outcomes in breast cancer.

Muliawan and Kin-Wah Lee study on hepatocellular carcinoma (HCC) highlights the role of CSCs in tumor progression and treatment resistance. CSC markers like CD24, CD44, and CD133 are linked to poor prognoses. Key regulators such as long non-coding RNAs, microRNAs, and pathways like Wnt and Notch influence CSC behavior and contribute to an immunosuppressive TME. Cytokines, particularly IL-1, IL-6, and IL-8, facilitate communication between CSCs and the TME, promoting tumor growth and immune evasion. Chemokines like CXCL2 and CXCR4 are crucial for metastasis, while growth factors such as TGF-β and VEGF drive tumor development.

In conclusion, liver CSCs play a crucial role in therapeutic resistance and tumor relapse in HCC patients by creating an immunosuppressive TME. CSCs secrete various immunosuppressive factors and interact with TME components. Targeting these factors is vital to counteract the TME, therefore further research is needed to develop effective immunotherapeutic strategies to enhance outcomes for HCC patients.

Luo et al. conducted a Mendelian randomization study exploring the causal relationships between 41 circulating cytokines and lung cancer risk. They identified four inflammatory mediators—SCF, IL-1β, IL-18, and IP-10—that may influence susceptibility to lung cancer, impacting overall risk and outcomes based on smoking status. While IL-1β promotes cancer progression through angiogenesis and immune suppression, IL-18 and IP-10 appear protective, enhancing immune responses against tumors.

The study’s limitations include insufficient statistical power and a sample from a single ethnicity, affecting generalizability. Overall, the findings suggest that these cytokine pathways could be potential targets for lung cancer prevention, detection, and treatment, highlighting their role in cancer susceptibility across various lung cancer types.

Elcheva et al. investigate the IGF2BP family of RNA binding proteins, known for promoting cell proliferation and tumor growth. This study reveals that inhibiting IGF2BPs enhances immune response genes, particularly through the type I and II interferon (IFN) signaling pathways. IGF2BP1 significantly influences interferon-stimulated genes, and its absence increases immune activity against tumors. In mouse models, reducing IGF2BP1 led to smaller tumors and greater immune cell infiltration. Combining IGF2BP1 inhibition with anti-PD-1 immunotherapy improved survival rates. IGF2BP1 and IGF2BP3 are absent in adult tissues but are reactivated in cancer like melanoma where they correlate with poorer outcomes, suggesting these proteins could be targeted for cancer therapies.

This research emphasizes IGF2BPs’ role in tumor progression and their potential as targets in enhancing immunotherapy effectiveness.

Overall, CSCs play a critical role in therapeutic resistance and tumor relapse across various cancers. Targeting their interactions within the TME, including the modulation of secretory factors and immune responses, is essential for improving patient outcomes. Continued research into these mechanisms and novel therapeutic strategies is vital for combating cancer effectively.

